# Sex-divergent anatomic system concordance in second primary malignant neoplasms: a large-scale analysis

**DOI:** 10.3389/fonc.2025.1720841

**Published:** 2026-01-09

**Authors:** Yadong Liu, Linping Shi, Wei Han, Xuejuan Duan, Pintian Lv, Na Li, Xianbo Zhang, Jinlong Liu, Jing Zhao

**Affiliations:** 1 Department of Oncology, Hebei General Hospital, Shijiazhuang, China; 2 Hebei General Hospital, Shijiazhuang, China; 3 Fourth Hospital of Hebei Medical University, Cancer Institute, Shijiazhuang, China; 4 Graduate School, Hebei North University, Hangjiakou, China

**Keywords:** association patterns, cancer combinations, multiple primary malignant neoplasms, second primary cancer, sex-based differences

## Abstract

**Objective:**

Using a second primary cancers (SPCs) database, this study aimed to analyze the epidemiological characteristics, systemic distribution, and correlation patterns of primary and SPCs across sexes. The goal was to identify potential sex-specific patterns and provide a reference for clinical prevention, diagnosis, and treatment.

**Methods:**

Clinical data of 918 patients with SPCs from two centers were retrospectively analyzed. Basic characteristics were summarized by sex. The systemic and specific type distributions of both primary and SPCs were analyzed. Association analyses explored cancer combination patterns and the tendency for subsequent cancers within the same system.

**Results:**

The cohort included 455 males (49.6%) and 463 females (50.4%), showing a balanced sex distribution (χ² = 0.143, *P* = 0.705). Primary cancers were predominantly located in the respiratory (298 cases), digestive (252 cases), and reproductive (161 cases) systems. SPCs were also most frequent in the respiratory (369 cases) and digestive (266 cases) systems. Stratified by sex, females exhibited higher proportions of cancers in the respiratory and reproductive systems for both primary cancer and SPC, whereas males showed a pronounced predominance in the digestive system. The top five primary cancer types were lung cancer (298 cases), breast cancer (117 cases), colorectal cancer (85 cases), thyroid cancer (60 cases), and esophageal cancer (54 cases). The top five SPCs were lung cancer (369 cases), colorectal cancer (108 cases), breast cancer (75 cases), thyroid cancer (61 cases), and gastric cancer (44 cases). Association analysis showed that 481 patients (52.4%) developed their SPC in the same organ system as their primary cancer, slightly more than the 437 patients (47.6%) with SPCs in different systems. Common cancer combination patterns included respiratory→respiratory (222 cases), digestive→digestive (147 cases), and reproductive→reproductive (60 cases). Among patients with second primary lung cancer, the proportion of adenocarcinoma was higher in females (170 cases) than in males (101 cases), while the proportion of squamous cell carcinoma was higher in males (53 cases) than in females (12 cases), indicating a statistically significant sex-related difference in pathological type distribution (*P* < 0.05).

**Conclusion:**

This large-sample study systematically reveals sex-based differences in the characteristics and associative patterns of SPCs. This study systematically defines sex-specific, high-risk cancer-system profiles (e.g., digestive-to-digestive in males, respiratory-to-respiratory and reproductive-to-reproductive in females) and establishes ‘anatomic system concordance’ as a fundamental principle in the development of SPCs. These findings provide a robust foundation for sex-specific and primary cancer type-guided surveillance strategies. Future clinical practice can utilize these findings to develop stratified management plans based on patient sex and initial primary cancer type, aiming to reduce the incidence and mortality of multiple primary malignant neoplasms.

## 1 Introduction

### 1.1 Background and significance

With advancements in medical technology, extended survival of cancer patients, and the widespread use of imaging diagnostics, the detection rate of second primary cancers(SPCs) is increasing annually, becoming a significant concern in clinical oncology (1). SPCs refer to the occurrence of a second, independent primary malignant tumor in the same patient at a different time and location. Their pathogenesis is complex, potentially involving genetic factors, shared carcinogenic exposures (e.g., smoking, alcohol consumption, environmental pollution), treatment-related carcinogenesis risks (e.g., radiotherapy toxicity, chemotherapy), and physiological structural differences, among other factors (2).

Current research on SPCs often focuses on single cancer combinations or small-sample analyses, lacking systematic investigation into the clinical characteristics, sex-based differences, and cancer association patterns in large patient cohorts. Due to differences in physiological structure, hormone levels, and lifestyle habits (e.g., higher rates of smoking and alcohol consumption in males, unique risks for reproductive system diseases in females), the distribution characteristics and patterns of multiple primary cancers may differ significantly between sexes. Furthermore, clarifying the association patterns between primary and SPCs, particularly the tendency for cancers within the same system, is crucial for developing individualized monitoring strategies and enabling early detection of SPCs.

Additionally, lung cancer, being a common type of SPC, exhibits sex-based differences in pathological types (e.g., adenocarcinoma, squamous cell carcinoma) which may reflect the influence of different pathogenic mechanisms and guide treatment selection. Based on this, this study retrospectively analyzes clinical data from 918 patients with SPCs across two centers to systematically investigate these issues, aiming to provide evidence-based support for the clinical management of multiple primary malignant neoplasms (MPMNs).No AI has been used in this article (3). This cohort/cross-sectional/case-control study has been reported in line with the STROCSS guidelines (4).

## 2 Materials and methods

### 2.1 Study population

This study included 918 patients diagnosed with SPCs during hospitalization at Hebei general Hospital and Fourth Hospital of Hebei Medical University between May 2019 and May 2025. Inclusion criteria were: 1) Patients with a previous pathological diagnosis of a malignant tumor, who subsequently received a pathological diagnosis of a SPC. 2) Exclusion of metastatic cancer, recurrent cancer, and multi-site lesions caused by tumor invasion. 3) Availability of complete clinical data, including hospitalization number, sex, date of birth, systemic classification and specific types of primary and SPCs, pathological types, etc. 4) Written informed consent was obtained from the patients for publication of their report and accompanying images. Exclusion criteria were: 1) Patients with a history of more than two primary malignant tumors. 2) Simultaneously diagnosed multifocal lesions of the same cancer type (e.g., multifocal colorectal cancer).

### 2.2 Data preprocessing

Clinical data were extracted from the electronic medical record systems of the two hospitals, initially involving 5,378 patient records related to SPCs. After excluding repeated hospitalizations, 918 unique patients were included in the analysis. Raw data underwent cleaning, organization, and coding processes, including handling missing values, standardizing data formats, and encoding categorical variables to ensure data quality and consistency.

The main variables collected for this study encompassed three categories: (1) basic demographic information, including sex and age (calculated from the date of birth); (2) primary cancer-related indicators, specifically the systemic classification (e.g., respiratory, digestive, reproductive, urinary, endocrine systems) and the specific tumor type (e.g., lung cancer, breast cancer, colorectal cancer); and (3) SPC-related indicators, which included its systemic classification, specific tumor type, and detailed pathological type (for lung cancer cases, encompassing adenocarcinoma, squamous cell carcinoma, and small cell carcinoma, among others).

Variable definitions referenced the International Classification of Diseases for Oncology, Third Edition (ICD-O-3). “Same system” was defined as both the primary and SPCs belonging to the same anatomical system (e.g., respiratory system includes lung, trachea, etc.; digestive system includes stomach, colorectum, esophagus, etc.).

We used the Surveillance, Epidemiology and End Results (SEER) Guidelines to define SPCs (5). According to the SEER definition of SPCs, the following criteria were used in our study: (1) tumors with ICD-O-3 histology codes that are different at the first, second or third number are SPCs; (2) one tumor characterized as “adeno-carcinoma, NOS” and another as a specific adenocarcinoma is regarded as a single tumor; (3) an invasive tumor following an *in situ* tumor >60 days after diagnosis is SPCs; (4) tumors with ICD-O-3 topography codes that are different at the second and/or third characters are multiple primaries; (5) tumors diagnosed >1 year apart are SPCs.

For certain special scenarios, laterality must be documented strictly in accordance with the SEER coding guidelines, and per the SEER Guidelines, the diagnosis of SPCs requires meeting both histologic coding and time interval criteria: For colorectal cancer, subsequent tumors occurring after radical resection, excluding anastomotic site lesions, are classified as SPCs after ruling out metastasis and recurrence; for lung cancers of the same histologic type, regardless of laterality, they are diagnosed as SPCs if the diagnosis interval exceeds 2 months (after excluding metastasis), while for tumors of the same histologic type in different lobes of the ipsilateral lung with a diagnosis interval ≤ 2 months, SPC diagnosis requires excluding not only recurrence/metastasis but also contiguous growth; for breast cancer, tumors in different quadrants or non-contiguous sites of the ipsilateral breast with a diagnosis interval ≤ 2 months are classified as SPCs if the histologic types are different, or if the histologic types are the same but sites are discrete and no evidence of metastasis exists, and new breast tumors occurring > 2 months after the initial diagnosis (regardless of histologic consistency) must exclude recurrence through evidence including a novel location, no regional lymph node or distant metastasis, and distinct histologic or molecular characteristics from the initial tumor; for endometrial cancer (EC) and ovarian cancer (OC), if the diagnosis interval is ≤ 2 months, they are classified as SPCs if EC is confined to the endometrium or myometrium, OC presents as an isolated ovarian lesion, and no contiguous invasion exists (with independence further verified by grade and molecular characteristics if both are endometrioid carcinomas), while if the diagnosis interval exceeds 2 months, OC diagnosed after EC requires confirmation as a new ovarian lesion without pelvic lymph node or distant metastasis, and EC diagnosed after OC requires confirmation as a primary endometrial lesion with no history of uterine invasion by OC.

### 2.3 Statistical analysis

Data processing and statistical analysis were performed using Python 3.9 and SPSS 26.0 software. Categorical data were presented as counts (n) and percentages (%). Frequency analysis was used to describe the basic characteristics of patients by sex and the systemic and type distributions of primary and SPCs. The Chi-square test was used to analyze sex-based differences in the pathological types of SPCs that were lung cancer. Cross-tabulation analysis was employed to explore cancer combination patterns between primary and SPCs, and the proportion of same-system occurrences was calculated. The significance level was set at α=0.05, with *P* < 0.05 considered statistically significant.

### 2.4 Visualization methods

Matplotlib 3.7 was used to generate bar charts (showing the systemic and type distributions of primary and SPCs by sex) and pie charts (showing the proportion of same-system vs. different-system occurrences for primary and SPCs) to visually present data distribution characteristics.

## 3 Results

### 3.1 Basic characteristics by sex

**Figure 1:**
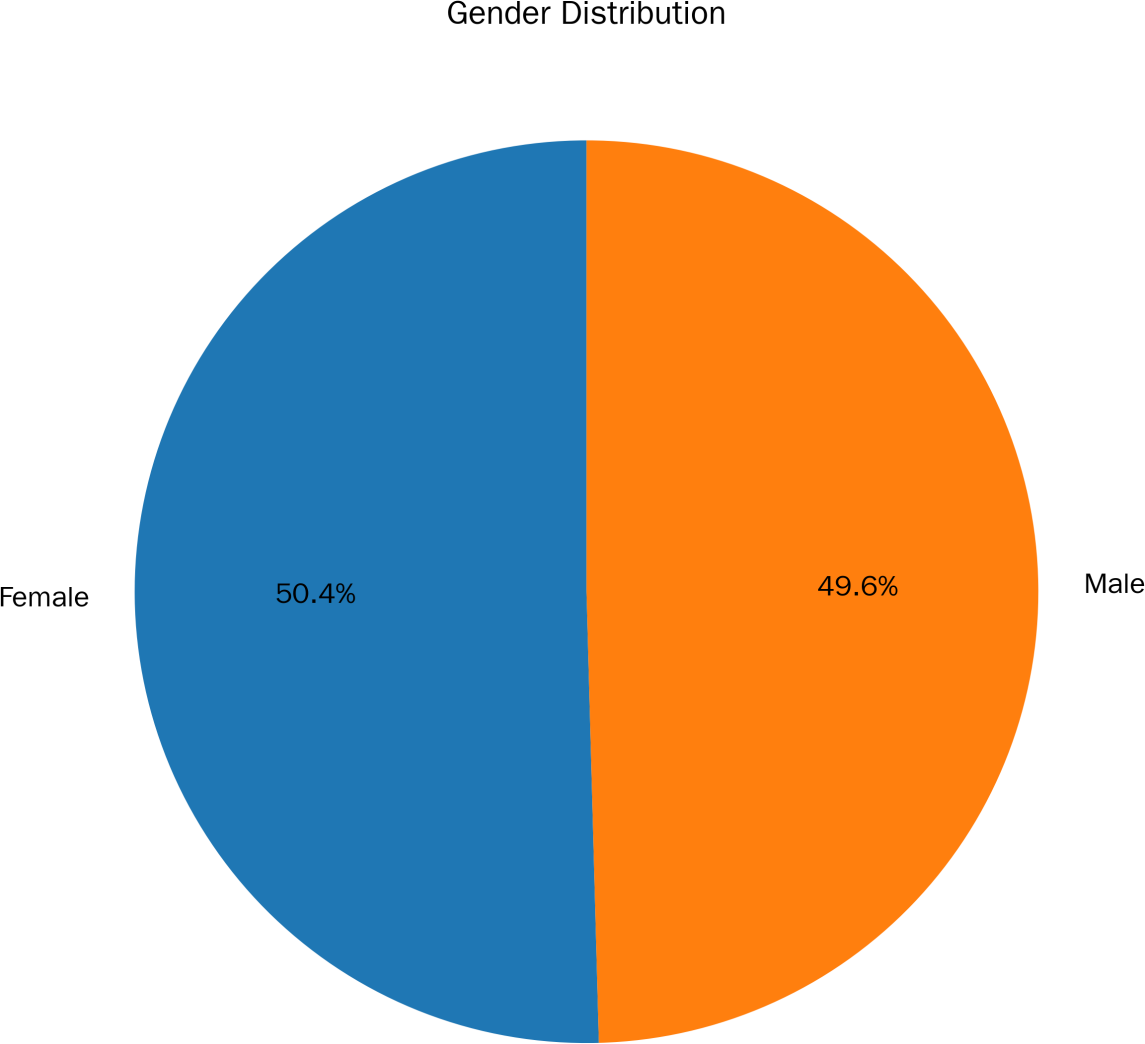
Gender distribution of patients with second primary cancer sex distribution of patients with second primary cancers (SPCs). Males accounted for 49.6% (n = 455) and females for 50.4% (n = 463).

Sex distribution: Males constituted approximately 49.6% (n = 455) and females 50.4% (n = 463), indicating a roughly balanced sex distribution (χ² = 0.143, P = 0.705) (**Figure 1**).

### 3.2 Type and systemic distribution of primary cancers by sex

#### 3.2.1 Top 5 systemic distributions of primary cancers (overall)

**Figure 2:**
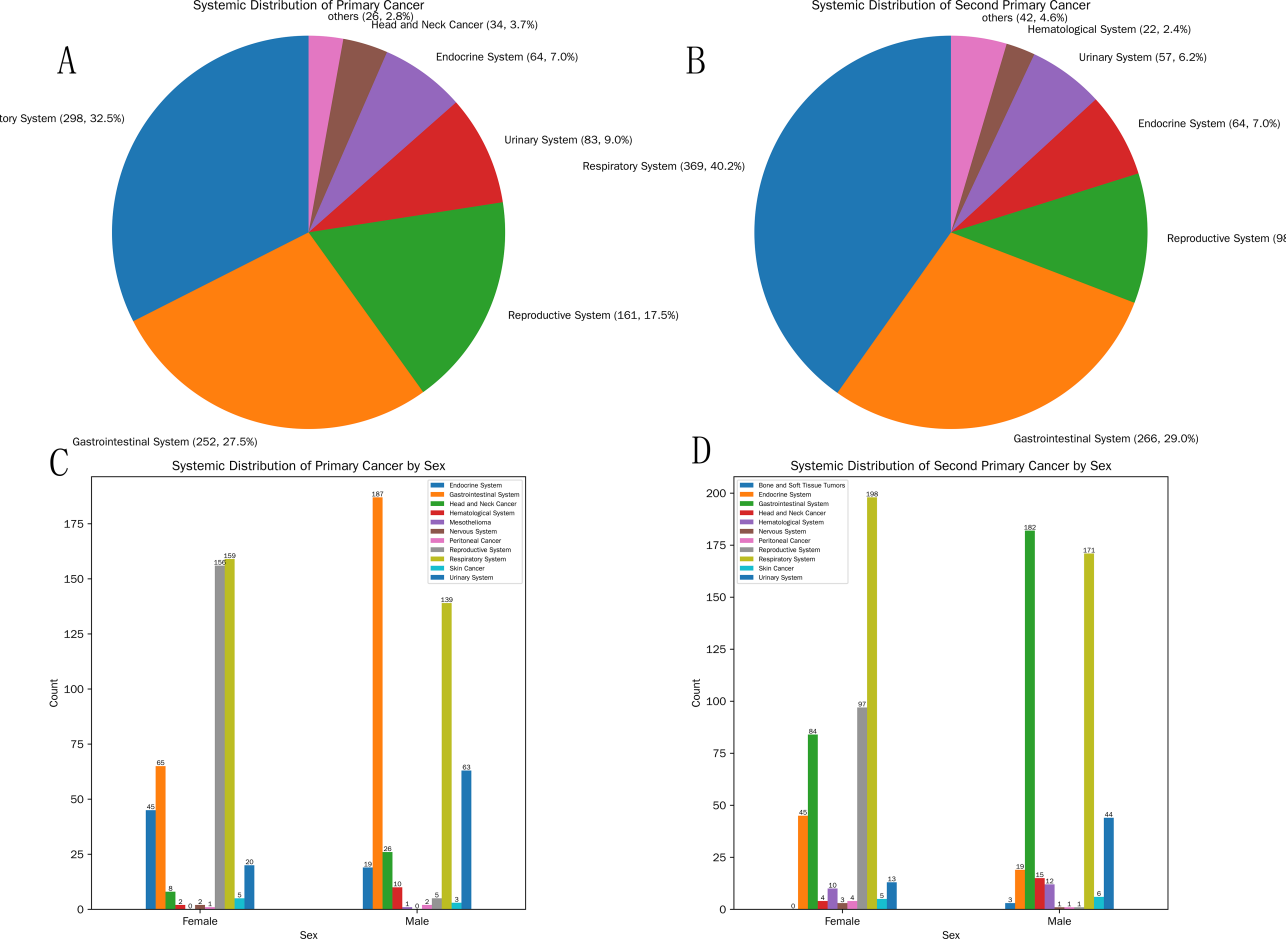
Systemic distribution of primary cancer and second primary cancer. **(A)** Overall systemic distribution of primary cancer; **(B)** Systemic distribution of second primary cancer; **(C)** Systemic distribution of primary cancer by gender group; **(d)** Systemic distribution of second primary cancer by gender group.

In the overall statistics for primary cancers, the respiratory system ranked first with 298 cases, making it the most frequent system. The digestive system followed closely with 252 cases. The reproductive system was third with 161 cases. The urinary and endocrine systems ranked fourth and fifth with 83 and 64 cases, respectively (**Figure 2A**).

#### 3.2.2 Top 5 systemic distributions of primary cancers by sex

1. Females: Among females, the respiratory system ranked first in primary cancer distribution with 159 cases. The reproductive system, with 156 cases, was very close, ranking second. The digestive, endocrine, and urinary systems ranked third, fourth, and fifth with 65, 45, and 20 cases, respectively.
2. Males: Among males, the digestive system had the highest number of primary cancers, reaching 187 cases. The respiratory system ranked second with 139 cases. The urinary system, head and neck cancers, and the endocrine system ranked third, fourth, and fifth with 63, 26, and 19 cases, respectively (**Table 1**; **Figure 2C**).

**Table 1:** Systemic distribution of primary cancers by gender (number of cases).

System	Female	Male
Respiratory System	159	139
Reproductive System	156	5
Gastrointestinal System	65	187
Endocrine System	45	19
Urinary System	20	63
Head and Neck Cancer	8	26
Skin Cancer	5	3
Peritoneal Cancer		2
Hematological System	2	10
Nervous System	2	0
Mesothelioma	0	1

#### 3.2.3 Specific type distribution of primary cancers

**Figure 3:**
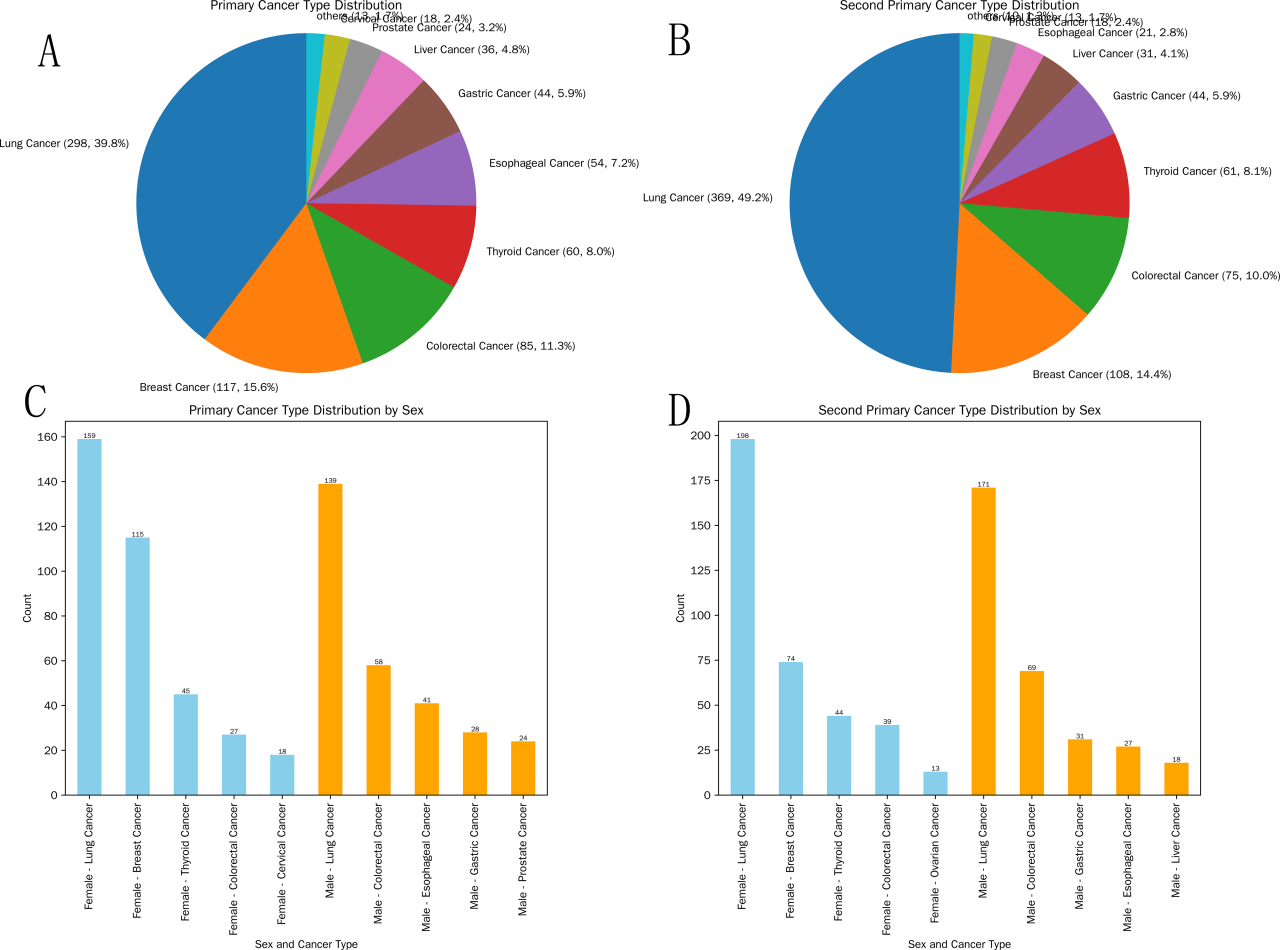
Distribution of cancer types in primary cancer and second primary cancer. **(A)** Overall distribution of cancer types in primary cancer; **(B)** Distribution of cancer types in second primary cancer; **(C)** Distribution of cancer types in primary cancer by gender group; **(D)** Distribution of cancer types in second primary cancer by gender group.

Top 5 primary cancer types: Lung cancer (298); Breast cancer (117); Colorectal cancer (85); Thyroid cancer (60); Esophageal cancer (54) (**Figure 3A**).

#### 3.2.4 Top 5 specific types of primary cancers by sex

Females: Lung cancer (159); Breast cancer (115); Thyroid cancer (45); Colorectal cancer (27); Cervical cancer (18).

Males: Lung cancer (139); Colorectal cancer (58); Esophageal cancer (41); Gastric cancer (28); Prostate cancer (24) (**Figure 3C**).

### 3.3 Type and systemic distribution of second primary cancers by sex

#### 3.3.1 Top 5 systemic distributions of second primary cancers (overall)

Among SPC systems, the respiratory system also occupied the first position, with a count of 369, significantly higher than other systems. The digestive system ranked second with 266 cases. The reproductive, endocrine, and urinary systems ranked third, fourth, and fifth with 98, 64, and 57 cases, respectively (**Figure 2B**).

#### 3.3.2 Top 5 systemic distributions of second primary cancers by sex

1. Females: For females, in the systemic distribution of SPCs, the respiratory system ranked first with 198 cases. The reproductive and digestive systems ranked second and third with 97 and 84 cases, respectively. The endocrine and urinary systems ranked fourth and fifth with 45 and 13 cases, respectively.
2. Males: For males, in the systemic distribution of SPCs, the digestive system ranked first with 182 cases. The respiratory system followed closely with 171 cases. The urinary system, endocrine system, and head and neck cancers ranked third, fourth, and fifth with 44, 19, and 15 cases, respectively (**Table 2**; **Figure 2D**).

**Table 2:** Systemic distribution of second primary cancers by gender (number of cases).

System	Female	Male
Respiratory System	198	171
Reproductive System	97	1
Gastrointestinal System	84	182
Endocrine System	45	19
Urinary System	13	44
Head and Neck Cancer	4	15
Skin Cancer	5	6
Peritoneal Cancer	3	1
Hematological System	10	12
Nervous System	3	1
Musculoskeletal System	0	3

#### 3.3.3 Specific type distribution of second primary cancers

Top 5 SPC types (overall): Lung cancer (369); Colorectal cancer (108); Breast cancer (75); Thyroid cancer (61); Gastric cancer (44) (**Figure 3B**).

#### 3.3.4 Top 5 specific types of second primary cancers by sex

Females: Lung cancer (198); Breast cancer (74); Thyroid cancer (44); Colorectal cancer (39); Ovarian cancer (13).

Males: Lung cancer (171); Colorectal cancer (69); Gastric cancer (31); Esophageal cancer (27); Liver cancer (18) (**Figure 3D**).

### 3.4 Association analysis between primary and second primary cancers

#### 3.4.1 Common cancer combination patterns

**Figure 4:**
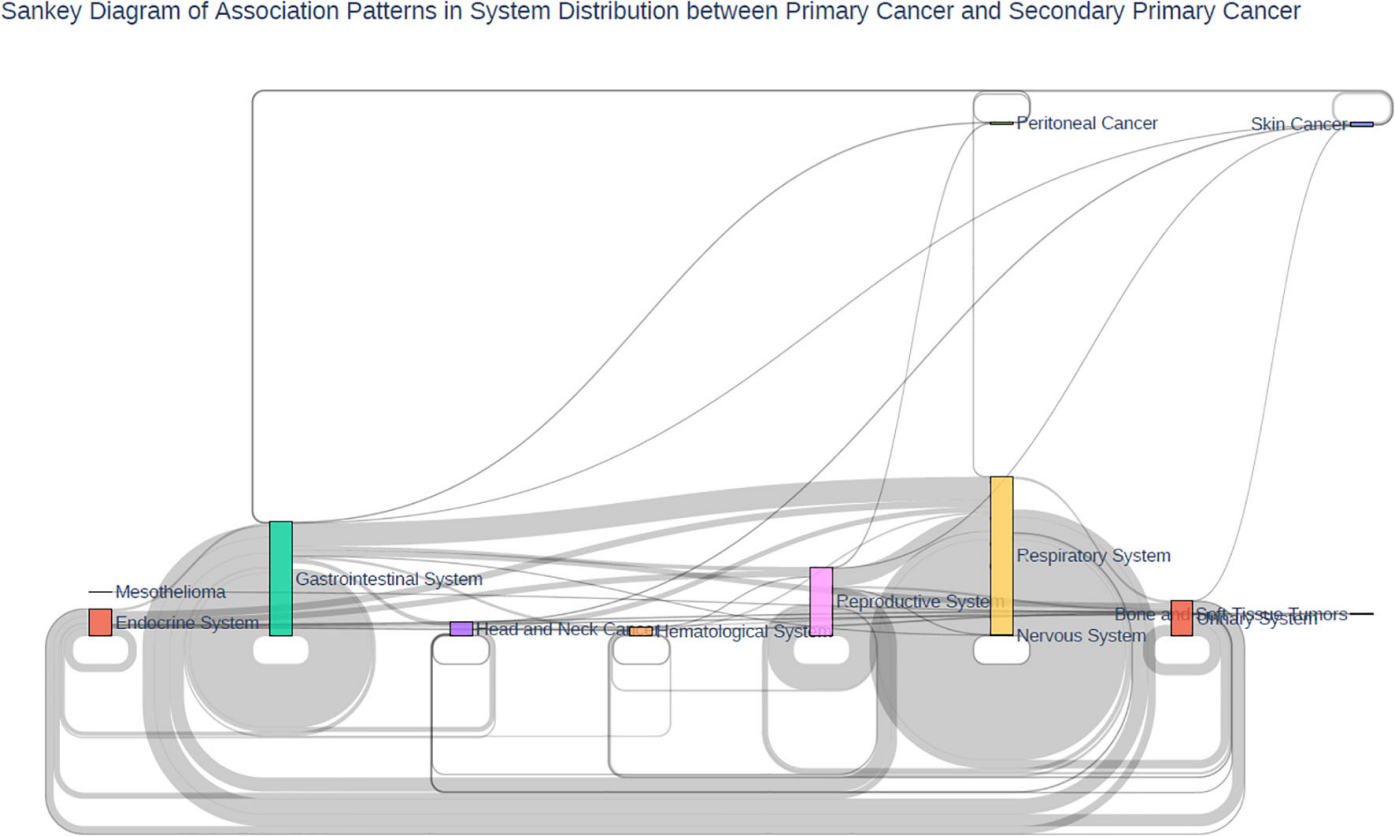
Sankey diagram of association patterns in system distribution between primary cancer and secondary primary cancer. By system (overall), the top three combination patterns were: Respiratory system → Respiratory system (222 cases); Digestive system → Digestive system (147 cases); Reproductive system → Reproductive system (60 cases); followed by Digestive system → Respiratory system (54 cases); Reproductive system → Respiratory system (41 cases); Respiratory system → Digestive system (36 cases).

By system (overall), the top three combination patterns were: Respiratory system → Respiratory system (222 cases); Digestive system → Digestive system (147 cases); Reproductive system → Reproductive system (60 cases); followed by Digestive system → Respiratory system (54 cases); Reproductive system → Respiratory system (41 cases); Respiratory system → Digestive system (36 cases). This indicates a high degree of systemic consistency between primary and SPCs (**Table 3**; **Figure 4**).

**Table 3:** Statistical table of the number of primary cancer combinations across different systems (number of cases).

Primary cancer	Second primary cancer	Number
Respiratory System	Respiratory System	222
Gastrointestinal System	Gastrointestinal System	147
Reproductive System	Reproductive System	60
Gastrointestinal System	Respiratory System	54
Reproductive System	Respiratory System	41
Respiratory System	Gastrointestinal System	36
Reproductive System	Gastrointestinal System	32
Urinary System	Gastrointestinal System	28
Urinary System	Urinary System	26
Endocrine System	Endocrine System	19

#### 3.4.2 Count statistics for same-system vs. different-system occurrences

**Figure 5:**
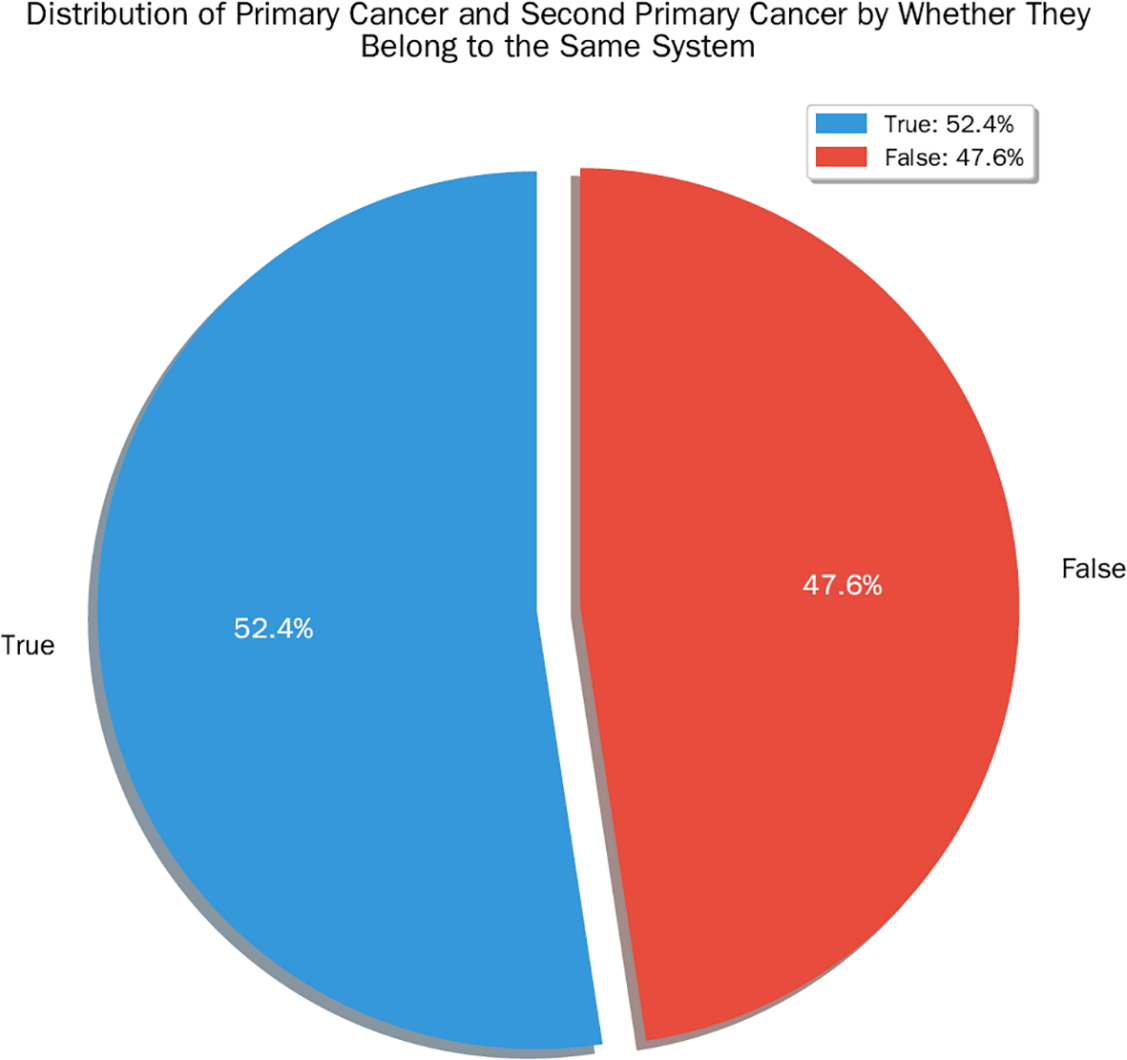
distribution of primary cancer and second primary cancer by whether they belong to the same system. The occurrence of SPCs within the same organ system as the primary cancer (481 cases, 52.4%) was statistically significantly more frequent than occurrence in a different system (437 cases, 47.6%; binomial test, *P* = 0.047).

The occurrence of SPCs within the same organ system as the primary cancer (481 cases, 52.4%) was statistically significantly more frequent than occurrence in a different system (437 cases, 47.6%; binomial test, *P* = 0.047). A binomial test (Z = 1.987, P = 0.047) indicated that this difference was statistically significant (**Figure 5**).

#### 3.4.3 Most common cancer combination patterns by sex

In males, common combinations were Digestive → Digestive (114 cases); Respiratory → Respiratory (97 cases); Digestive → Respiratory (38 cases). In females, common combinations were Respiratory → Respiratory (125 cases); Reproductive → Reproductive (59 cases); Reproductive → Respiratory (39 cases).

This distribution table shows that when the respiratory system was the site of the primary cancer, the number of second primary cancers also occurring in the respiratory system was much higher in females (125.0) than in males (97.0). Conversely, when the digestive system was the site of the primary cancer, the number of second primary cancers also occurring in the digestive system was significantly higher in males (114.0) than in females (33.0). Regarding the reproductive system, the number of cases where both primary and second primary cancers occurred within the reproductive system was far higher in females (59.0) than in males (1.0) (**Table 4**).

**Table 4:** Corresponding table of systemic distribution between primary cancer and second primary cancer (number of cases).

Sex	Primary cancer	Endocrine	Respiratory	Head and neck	Urinary	Gastrointestinal	Reproductive	Skin	Nervous	Peritoneal	Hematological	Bone and soft tissue
F	Endocrine	13	10	1	1	4	14	0	0	0	2	0
F	Respiratory	13	125	0	0	9	9	0	0	0	3	0
F	Head and Neck	1	3	1	0	3	0	0	0	0	0	0
F	Urinary	1	4	1	6	3	5	0	0	0	0	0
F	Gastrointestinal	4	16	0	1	33	9	0	0	0	1	0
F	Reproductive	13	39	1	5	31	59	1	1	2	4	0
F	Skin	0	0	0	0	1	0	4	0	0	0	0
F	Nervous	0	0	0	0	0	0	0	2	0	0	0
F	Peritoneal	0	0	0	0	0	0	0	0	1	0	0
F	Hematological	0	1	0	0	0	1	0	0	0	0	0
M	Endocrine	6	6	2	1	2	0	0	0	0	0	2
M	Respiratory	5	97	2	6	27	0	0	0	0	2	0
M	Head and Neck	2	9	1	3	8	0	2	0	0	1	0
M	Urinary	0	15	0	20	25	0	1	0	0	2	0
M	Gastrointestinal	5	38	10	12	114	0	1	1	1	5	0
M	Reproductive	1	2	0	0	1	1	0	0	0	0	0
M	Skin	0	1	0	0	0	0	2	0	0	0	0
M	Peritoneal	0	0	0	0	2	0	0	0	0	0	0
M	Hematological	0	3	0	2	3	0	0	0	0	2	0
M	Mesothelioma	0	0	0	0	0	0	0	0	0	0	1

### 3.5 Subgroup analysis

Among patients whose SPC was lung cancer, the distribution of pathological types differed by sex. For instance, the number of adenocarcinomas was significantly higher in females (170) than in males (101), while the number of squamous cell carcinomas was higher in males (53) than in females (12). The Chi-square test (χ² = 28.735, P < 0.001) indicated a statistically significant difference in the pathological types of the second primary lung cancer between sexes. This result suggests a significant association between the pathological type of the second primary lung cancer and patient sex, highlighting the importance of considering sex factors in the diagnosis, treatment, and research of lung cancer.

When the primary cancer was breast cancer, the most frequent SPC was again breast cancer (37 cases). When the primary cancer was esophageal cancer, the most frequent SPC was lung cancer (13 cases). For patients with primary bladder cancer, the most frequent SPC was colorectal cancer (5 cases).

The high number of second primary breast cancers following a primary breast cancer might suggest that breast cancer patients possess intrinsic factors that increase their risk of developing a second breast cancer. Lung cancer also featured prominently in this group. In cases where the primary cancer was esophageal cancer, lung cancer was the most common SPC.

Overall, the distribution of common types and counts of SPCs varied significantly depending on the type of primary cancer.

## 4 Discussion

Among 918 patients with SPCs, this study found a nearly equal sex distribution (49.6% male; 50.4% female), consistent with some previous reports indicating a slightly higher detection rate of multiple primary malignant neoplasms (MPMNs) in females (5). This may be related to longer average life expectancy in women, higher screening rates for hormone-sensitive organ cancers like breast and thyroid, and potentially the interplay of genetic susceptibility, hormone levels, and lifestyle factors. Study indicate that certain gene mutations (e.g., BRCA1/2) are closely associated with the risk of specific cancers in women (6), which may further influence MPMN incidence. Additionally, Longer exposure time to endogenous hormones, such as a later age at natural menopause (≥55 years), longer reproductive years (more ovulatory cycles), and a higher number of births (≥5 births), are significantly associated with an increased risk of thyroid cancer in postmenopausal women (7). Additionally, cancers like breast and thyroid often have better prognoses and longer survival, allowing more time for a SPC to develop, explaining the higher proportion of primary cancers in the reproductive and endocrine systems in females compared to males.

Building on the overall distribution pattern, This study found that both primary and SPCs were predominantly distributed in the respiratory and digestive systems, underscoring their central role in carcinogenesis. This is closely linked to the physiological functions of these systems and their exposure to carcinogens. The respiratory system is directly exposed to environmental pollutants and tobacco smoke, making it a high-risk site for malignancies like lung cancer (8). The digestive system is chronically exposed to dietary carcinogens (e.g., nitrosamines, aflatoxins), and factors like H. pylori infection and chronic inflammation increase the risk of gastric cancers (9). The proportion of SPCs in the respiratory system (40.2%) was higher than that of primary cancers (32.5%). This may be attributed to advancements in lung cancer screening technologies (e.g., low-dose spiral CT), which have significantly improved detection rates (10). Furthermore, treatment for cancers at other sites, especially radiotherapy, can damage lung tissue, increasing the risk of SPCs (11). The lung is also a common site for metastasis, leading to more frequent surveillance during follow-up, which could also contribute to the higher detection rate of SPCs. Relevant literature supports these findings (12), highlighting the close relationship between digestive system cancers and environmental/lifestyle factors, as well as the association of lung cancer with secondhand smoke and air pollution. For instance, studies show gastric cancer is strongly linked to H. pylori infection and chronic gastritis, while colorectal cancer is associated with dietary habits, obesity, and other lifestyle factors (13).

Further stratification by sex revealed distinct association patterns. Females had significantly higher proportions of cancers in the respiratory (primary: 159; SPC: 198) and reproductive (primary: 156; SPC: 97) systems compared to males. This phenomenon may be linked to female-specific physiological factors and environmental influences. For example, estrogen not only plays a role in reproductive system tumors but may also affect lung tissue repair mechanisms, increasing the risk of respiratory system tumors in women (14). Furthermore, the high incidence of common female reproductive system cancers like breast and cervical cancer accentuates this sex disparity. Conversely, males showed a predominance of digestive system tumors (primary: 187; SPC: 182), closely associated with lifestyle factors. Higher prevalence of smoking and alcohol consumption, coupled with unhealthy dietary habits like high salt and fat intake, increase the risk of digestive system cancers in men. The incidence of specific digestive cancers like gastric and liver cancer is consistently higher in males across different regions and populations. Therefore, cancer prevention and treatment strategies should consider these biological and socio-cultural factors for more effective intervention and management, such as enhanced lung and reproductive system cancer screening for female cancer survivors, and health education and lifestyle interventions focused on digestive system cancers for male patients.

For specific cancer types, lung, breast, and colorectal cancers consistently ranked among the top three for both primary cancers and SPCs. Lung cancer, being highly prevalent as both a primary cancer and SPCs, is associated with universal risk factors like smoking and air pollution. It may also be that extended survival of lung cancer patients, coupled with the use of chemotherapeutic agents and a history of thoracic radiotherapy, increases the risk of developing other primary cancers (15), notably colorectal and breast cancer. Breast cancer, a highly prevalent female-specific cancer, remained the third most common SPC, indicating that breast cancer survivors require long-term monitoring for new tumors in the breast and other sites. The necessity for long-term monitoring for recurrence and new cancers in breast cancer survivors is widely recognized, particularly among younger women (16). According to a multicenter study (17), breast cancer patients have a significantly increased risk of developing SPCs after treatment, with notably higher incidences of colorectal and lung cancer compared to the general population. A retrospective study of cancer patients showed that the probability of developing an SPC within 5 years after treatment was 8.2%, rising to 13.9% within 10 years (18).

Further sex stratification again revealed distinct patterns. The proportions of breast cancer (115 cases) and thyroid cancer (45 cases) among primary caner were far higher in females than males, closely related to changes in female hormone levels and their effects on breast and thyroid tissue. In contrast, males had higher proportions of colorectal cancer (58 cases), esophageal cancer (41 cases), and prostate cancer (24 cases) among primary caner, directly related to male physiological characteristics and habits. Prostate cancer is a male-specific reproductive system tumor whose incidence rises significantly with age. The incidence rate is higher in European and American populations, while it is relatively lower in Asian populations (19). Compared with other highly prevalent tumors such as lung cancer and colorectal cancer, the overall incidence of prostate cancer in the population of the region where the research was conducted is relatively low, which may lead to its under-representation in this cohort. The high incidence of colorectal and esophageal cancers is associated with the generally higher rates of smoking and alcohol consumption in men, which can damage the digestive tract mucosa and increase cancer risk (20).

This study clearly identifies “same-system continuation” as a core characteristic of MPMN associations, with 52.4% of patients developing their SPC within the same organ system as their primary cancer, slightly higher than different-system occurrences, consistent with findings in existing literature (21). Common combinations were respiratory→respiratory, digestive→digestive, and reproductive→reproductive. This phenomenon may stem from several mechanisms: (1) Organs within the same system share similar cell types and physiological environments, leading to consistent susceptibility to common carcinogenic factors (e.g., tobacco smoke for the respiratory system, dietary carcinogens for the digestive system). (2) Tumor treatment-related damage, such as chest radiotherapy increasing the risk of second primary lung cancer, or gastrointestinal surgery/chemotherapy inducing abnormal mucosal hyperplasia. (3) Genetic factors or aberrant molecular pathways, where certain gene mutations might simultaneously affect cell proliferation and apoptosis regulation in multiple organs of the same system. Respiratory→respiratory (222 cases) was the most common combination, with lung→lung being predominant. One study noted that thoracic radiotherapy significantly increases the risk of second primary lung cancer (22), aligning with our findings and suggesting that lung cancer survivors, especially long-term smokers or those with a family history, require enhanced pulmonary monitoring. Genetic factors also play a crucial role in MPMN development. Research indicates that specific gene mutations are closely associated with MPMNs, particularly in digestive and reproductive system tumors (23). This study also found that within the digestive→digestive combination (147 cases), colorectal→gastric and gastric→colorectal cancers were relatively common, potentially linked to shared pathogenic factors like chronic gastrointestinal inflammation and H. pylori infection. Clinically, patients with a history of one digestive system cancer might benefit from cross-site screening during follow-up (e.g., regular gastroscopy for colorectal cancer survivors).

The reproductive→reproductive combination (60 cases) was primarily observed in females (59 cases), mainly involving breast→ovarian and breast→cervical cancer patterns. This may be associated with specific genetic mutations in females. For example, the relevance of BRCA gene mutations to SPCs is gaining attention, especially in female patients where breast and ovarian cancers are closely linked to these mutations. Research shows BRCA1 and BRCA2 play vital roles not only in DNA repair and homologous recombination but also in germ cell development and function, potentially explaining how these mutations influence cancer risk (24). A prospective study (25) found that women carrying BRCA mutations showed decreased ovarian reserve and accumulated oocyte DNA damage even before showing signs of cancer, further emphasizing the importance of regular genetic testing and reproductive system monitoring. Patients with reproductive system tumors and BRCA mutations warrant particularly close follow-up for SPCs. This may be also related to high BMI in females. A categorical meta-analysis on BMI, incorporating 13 prospective studies (5 cohorts, 8 nested case-control studies), showed obesity was significantly associated with increased risk of contralateral breast cancer (RR = 1.37, 95%CI: 1.20-1.57), breast cancer SPCs (RR = 1.40, 95%CI: 1.24-1.58), endometrial cancer (RR = 1.96, 95%CI: 1.43-2.70), and colorectal cancer (RR = 1.89, 95%CI: 1.28-2.79) as SPCs. Dose-response meta-analysis indicated a 12% (RR = 1.12, 95%CI: 1.06-1.20) and 14% (RR = 1.14, 95%CI: 1.07-1.21) increased risk for contralateral breast cancer and any SPC, respectively, per 5 kg/m² increase in BMI. The pooled RR for endometrial SPC was 1.46 (95%CI: 1.17-1.83) per 5-unit increase (26). This underscores the importance of preventive policies aimed at reducing overweight and obesity rates. Clinical trials evaluating the impact of weight normalization on SPC incidence in overweight breast cancer survivors are needed. In future studies, we will incorporate this issue and conduct in-depth analyses.

Among patients with SPC lung cancer, the proportion of adenocarcinoma was significantly higher in females (170 cases) than males (101 cases), while the proportion of squamous cell carcinoma was higher in males (53 cases) than females (12 cases). This aligns with the established consensus on sex differences in lung cancer pathology (27). The higher incidence of adenocarcinoma in females may be related to estrogen-mediated cell proliferation, exposure to secondhand smoke (which is more likely to induce adenocarcinoma), and genetic mutations (e.g., higher EGFR mutation rates). The higher incidence of squamous cell carcinoma in males is closely associated with long-term smoking causing bronchial mucosal squamous metaplasia (28, 29).

In summary, this systematic analysis demonstrates no sex difference in the incidence rate of SPCs within this cohort. However, the systemic and type distributions of cancers exhibited sex-specific patterns, reflecting physiological and risk exposure differences. System-wise, the respiratory and digestive systems are core sites for MPMNs, but with significant sex disparities. Regarding specific cancer types, lung cancer was the most common primary and SPC. The cancer association pattern highlights “systemic consistency,” with distinct sex-specific dominant associations: males primarily showed “digestive→digestive,” followed by “respiratory→respiratory”; females primarily showed “respiratory→respiratory” and “reproductive→reproductive.” In the subgroup with SPC lung cancer, the sex difference in pathological types holds clinical value: adenocarcinoma was significantly more common in females, while squamous cell carcinoma was significantly more common in males.

Based on the genetic and clinical characteristics identified in this study, it is recommended that individualized screening and monitoring protocols be developed for cancer survivors to improve the early detection rate of secondary malignant tumors and optimize clinical outcomes: (1) Develop differentiated monitoring strategies based on patient sex: intensify screening for respiratory, reproductive, and endocrine systems in females; focus on digestive and urinary systems in males. (2) Implement targeted surveillance based on common cancer combination patterns (e.g., regular lung CT scans for lung cancer survivors; regular gastroscopy for colorectal cancer survivors). (3) In the early screening of second primary lung cancer, gender differences can be considered to optimize imaging evaluation and pathological biopsy strategies. For example, more attention should be paid to imaging features related to adenocarcinoma in female survivors, and vigilance for squamous cell carcinoma screening should be enhanced in male long-term smoking survivors.

## 5 Study limitations and future directions

This dual-center retrospective analysis, despite its large sample size, may still be subject to selection bias. Data sources might have limitations, and the sample’s representativeness might not be broad enough, as characteristics may vary across regions and ethnic groups. This system-level analysis is exploratory and descriptive and may lack sufficient detail and epidemiological meaningfulness in relation to the heterogeneous etiologies of cancers within the same system. Future research should involve multicenter prospective studies, integrating genomic and epidemiological data to further clarify the causative factors and molecular mechanisms of SPCs, providing stronger evidence for precise prevention and treatment.

## Data Availability

The raw data supporting the conclusions of this article will be made available by the authors, without undue reservation.
